# Progressive multifocal leukoencephalopathy associated with systemic lupus erythematosus: longitudinal observation of lymphocytes, JC virus in cerebrospinal fluid, and brain magnetic resonance imaging

**DOI:** 10.1007/s13365-024-01203-0

**Published:** 2024-03-19

**Authors:** Hidetada Yamada, Megumi Toko, Masahiro Nakamori, Hiroki Ueno, Shiro Aoki, Tomohiro Sugimoto, Hiroko Yasutomi, Kazuo Nakamichi, Hirofumi Maruyama

**Affiliations:** 1 Department of Clinical Neuroscience and Therapeutics, Hiroshima University Graduate School of Biomedical and Health Sciences, Hiroshima, Japan; 2 Department of Neurology, Hiroshima City Hiroshima Citizens Hospital, Hiroshima, Japan; 3 Department of Clinical Immunology and Rheumatology, Hiroshima University Hospital, Hiroshima, Japan; 4 Department of Diagnostic Radiology, Hiroshima University Hospital, Hiroshima, Japan; 5 Department of Virology 1, National Institute of Infectious Diseases, Tokyo, Japan

**Keywords:** Progressive multifocal leukoencephalopathy, JC virus, Systemic lupus erythematosus, Lymphocytopenia, Immunosuppression therapy, Punctate lesions

## Abstract

Progressive multifocal leukoencephalopathy (PML) rarely occurs in patients with systemic lupus erythematosus (SLE). This report presents the case of a patient who developed PML due to SLE-associated multiple factors. A 60-year-old woman diagnosed with SLE undergoing multiple immunosuppressive therapies, including azathioprine, presented with cerebral cortical symptoms, lymphocytopenia, and vitamin B12 deficiency and was subsequently diagnosed with SLE-associated PML. We evaluated the cause and disease activity of PML, focusing on the longitudinal assessment of lymphocytopenia, JC virus (JCV) DNA copy number in the cerebrospinal fluid, and magnetic resonance imaging (MRI) findings. Discontinuing azathioprine and initiating alternative immunosuppressive treatments with intramuscular vitamin B12 injections affected lymphocytopenia and disease management. However, despite recovery from lymphopenia and JCV DNA copy number being low, the large hyperintense and punctate lesions observed on the fluid-attenuated inversion recovery (FLAIR) images exhibited varying behaviors, indicating that the balance between contributing factors for PML may have fluctuated after the initial treatment. Clinicians should be meticulous when assessing the underlying pathology of the multifactorial causes of PML due to SLE. The difference in the transition pattern of these lesions on FLAIR images may be one of the characteristics of MRI findings in PML associated with SLE, reflecting fluctuations in disease activity and the progression stage of PML.

## Introduction

Progressive multifocal leukoencephalopathy (PML) is a rare yet potentially fatal demyelinating disease of the central nervous system, which usually affects patients who are immunocompromised, including those with systemic lupus erythematosus (SLE) (Bernard-Valnet et al. 2021; Henegar et al. 2016). Management of PML depends on restoring immune system function, and evaluating its cause and disease activity is crucial due to different treatment approaches for PML associated with SLE (Bernard-Valnet et al. 2021). Therefore, clinicians should determine whether PML is resulted from immunosuppressive therapy or SLE-related complications. Recent advancements in magnetic resonance imaging (MRI) can support the assessment of disease progression of PML (Thurnher et al. 2019; Miyagawa et al. 2014). This report describes a case of PML due to SLE with lymphocytopenia. The cause and disease activity of PML were evaluated, focusing on longitudinal assessment of variable supportive parameters.

## Case report

A 60-year-old woman was diagnosed with SLE 17 years ago based on clinical features, such as polyarthritis and fever, and serological studies. The patient had a low disease activity state by initial treatment. One year after discontinuation of immunosuppressive therapy because of personal reasons, she experienced a relapse; hence, a daily regimen of immunosuppressive therapy comprising 10 mg of prednisolone (PSL), 50 mg of azathioprine (AZA), and 1.5 mg of tacrolimus (TAC) was resumed. However, the low-grade fever persisted until admission to our hospital.

The patient exhibited various symptoms, including agraphia, amnesic aphasia, acalculia, left–right agnosia, ideational apraxia, hemispatial neglect, and homonymous hemianopsia. Blood tests showed a low white blood cell count (1.56 × 10^3^/μL) with lymphocytopenia (360/μL; CD4^+^ lymphocytes 193/μL, CD8^+^ lymphocytes 164/μL), hypocomplementemia, elevated anti-ds DNA antibodies (37.7 IU/mL), and vitamin B12 deficiency (125 pg/mL). Anemia or thrombocytopenia was not observed. Antibody tests for cytomegalovirus, hepatitis B viruses, human immunodeficiency virus, and intrinsic factor antibodies were negative. Tuberculosis-interferon-gamma release assay results were negative. Gastric endoscopy was not performed. Bone marrow cells showed mild hyperplasia with normal maturation and differentiation without dysplasia. A cerebrospinal fluid (CSF) examination showed no white blood cells or malignant cells and a protein level of 63 mg/dL. The real-time polymerase chain reaction showed 758 copies/mL of JC virus (JCV) DNA in the CSF.

MRI revealed high-intensity signals in the bilateral parieto-occipital lobe white matter on fluid-attenuated inversion recovery (FLAIR) images, as well as multiple punctate lesions in the bilateral frontal lobe white matter. Susceptibility-weighted imaging (SWI) showed hypointense signal rims in a confined lesion in the left occipital lobe cortex adjacent to the white matter lesion. No discernible enhancement was evident on T1-weighted images following gadolinium administration. Based on these diagnostic findings, the patient was diagnosed with PML secondary to SLE. Subsequently, AZA was discontinued after admission.

**Fig. 1:**
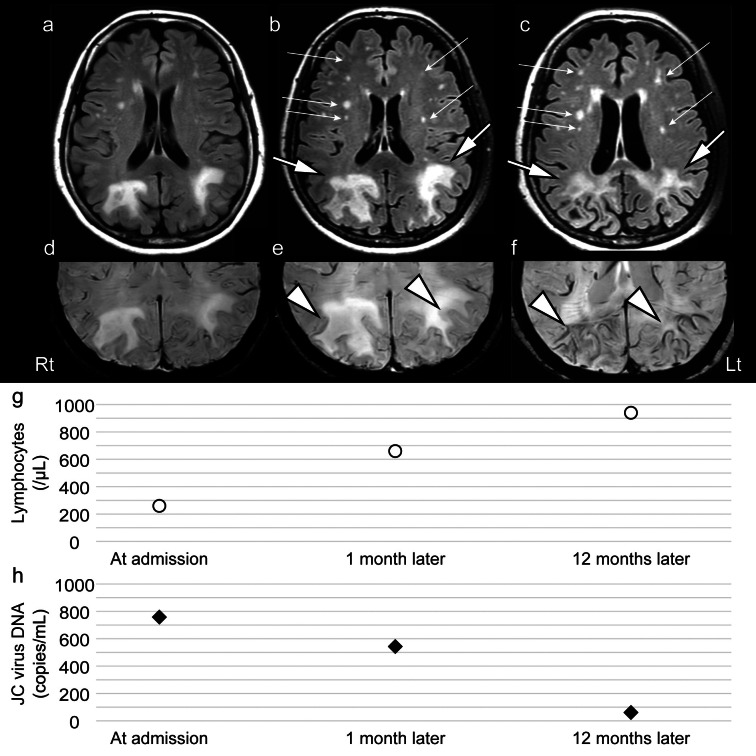
MRI and JC virus DNA copy numbers in the CSF at each time point are shown. Axial FLAIR images **a** at admission, **b** one month later, and **c** 12 months later are shown. SWI findings **d** at admission, **e** one month later, and **f** 12 months later are shown. Lymphocyte number **g** and JC virus DNA copy number in the CSF **h** at each time point are summarized. Both large hyperintense and punctate lesions are enlarged on FLAIR images (one month later). After 12 months, the initially large hyperintense lesions on FLAIR images gradually shrank and became pale (large arrows). The punctate lesions on FLAIR images remained prominent (small arrows). SWI shows hypointense signal rims adjacent to white matter lesions in the bilateral parieto-occipital lobe cortex, becoming more prominent over time (arrowheads). The follow-up lymphocyte counts were 660 and 940/μL at one and 12 months. The JC virus DNA copy number in the CSF significantly decreased from 543 to 61 copies/mL after 12 months. Abbreviations: MRI, magnetic resonance imaging; CSF, cerebrospinal fluid; FLAIR, fluid-attenuated inversion recovery; SWI, susceptibility-weighted imaging

Despite a low SLE disease activity index (SLEDAI) score of 5, the disease activity may have smoldered (Cook et al. 2000). Therefore, immunosuppressive therapy was administered, including PSL, TAC, and hydroxychloroquine (HCQ), in addition to mirtazapine, risperidone, mefloquine, and intramuscular injections of vitamin B12. Since her lymphocyte counts improved one month after therapy resumed, PSL was subsequently reduced. Follow-up MRI examinations showed large hyperintense and punctate lesions exhibiting varying behaviors on FLAIR images. The hypointense signal rims on SWI became more prominent (Fig. 1). Although cognitive abnormalities persisted, only ideational apraxia showed improvement, and the patient’s condition was stable. She continued to live in her own house with caregiver support for > 13 months.

## Discussion

Because PML can develop in patients with SLE contributing to multiple factors including the underlying illness and the treatment applied, the initial focus of this case should be assessing the balance between these factors (Henegar et al. 2016). The factors in this case included smoldering SLE disease activity, lymphocytopenia, and multiple immunosuppressive therapies on admission. The first step involved investigating the cause of lymphocytopenia, which was determined not to be due to an infection but rather drug- and immune-mediated causes, such as prolonged use of glucocorticoids and AZA, as well as lymphocytotoxic antibodies associated with SLE disease activity (Chen et al. 2023; Gómez-Martín et al. 2011; Li et al. 2014; Velo-García et al. 2016). Initial treatment, including discontinuing AZA and administering PSL, TAC, and HCQ, affected the lymphocytopenia, SLE, and PML disease activity. In addition, lymphocytopenia was also modulated by intramuscular vitamin B12 injections (Tamura et al. 1999). However, despite recovery from lymphopenia, the punctate lesions on FLAIR images remained prominent after 12 months. Although PSL was reduced to control the drug-associated factor after one month, the balance between contributing factors for PML may have fluctuated after the initial treatment.

Longitudinal changes in MRI findings could provide clinical implications for the patient’s clinical course. Punctate hyperintense lesions on T2-/FLAIR images are early signs of temporary lymphocytopenia-related PML caused by natalizumab and other drugs, different from those of HIV-PML (Hodel et al. 2015; Ueno et al. 2020; Ishii et al. 2018). Since the management of drug-associated PML follows a monophasic pattern relying on restoring the cellular immune response by tapering these drugs, both punctate and large hyperintense lesions on T2-/FLAIR images also follow a monophasic pattern. In contrast, the causes of PML with SLE are multifactorial. Therefore, the difference in the transition pattern of these lesions may be a characteristic MRI finding in PML associated with SLE, reflecting fluctuations in disease activity and progression stage of PML (Ono et al. 2019). In addition, the hypointense rim in the paralesional U-fibers on SWI not only is an early marker of PML but also appears or becomes more prominent in long-term survivors with an accumulation of iron in macrophages (Thurnher et al. 2019; Hodel et al. 2015; Mahajan et al. 2021). Therefore, hypointense rims on SWI accompanied with large hyperintense lesions on T2-/FLAIR became more prominent with cortical atrophy and low copies of JCV DNA (possibly indicating end-stage neuroinflammation), suggesting heterogeneity in disease modifications in PML in the present case (Thurnher et al. 2019).

In summary, the cause of SLE-associated PML can be multifactorial. Therefore, clinicians should be careful when assessing the causes and disease activity of PML. In this case, time-dependent changes in multiple aspects supported the assessment, including the number of lymphocytes, JCV copy number in the CSF, and MRI findings.

## Data Availability

No datasets were generated or analysed during the current study.
